# Synergistic Effect of Bioactive Inorganic Fillers in Enhancing Properties of Dentin Adhesives—A Review

**DOI:** 10.3390/polym13132169

**Published:** 2021-06-30

**Authors:** Imran Farooq, Saqib Ali, Samar Al-Saleh, Eman M. AlHamdan, Mohammad H. AlRefeai, Tariq Abduljabbar, Fahim Vohra

**Affiliations:** 1 Faculty of Dentistry, University of Toronto, Toronto, ON M5G 1G6, Canada; 2 Department of Biomedical Dental Sciences, College of Dentistry, Imam Abdulrahman Bin Faisal University, Dammam 31441, Saudi Arabia; drsaqiibali@gmail.com; 3 Prosthetic Dental Science, College of Dentistry, King Saud University, Riyadh 11545, Saudi Arabia; salsaleh@ksu.edu.sa (S.A.-S.); ealhamdan@ksu.edu.sa (E.M.A.); tajabbar@ksu.edu.sa (T.A.); fvohra@ksu.edu.sa (F.V.); 4 Operative Division, Department of Restorative Dentistry, College of Dentistry, King Saud University, Riyadh 11545, Saudi Arabia; malrefeai@ksu.edu.sa

**Keywords:** bioactive fillers, dentin adhesive, dental resin composite, remineralization

## Abstract

Dentin adhesives (DAs) play a critical role in the clinical success of dental resin composite (DRC) restorations. A strong bond between the adhesive and dentin improves the longevity of the restoration, but it is strongly dependent on the various properties of DAs. The current review was aimed at summarizing the information present in the literature regarding the improvement of the properties of DAs noticed after the addition of bioactive inorganic fillers. From our search, we were able to find evidence of multiple bioactive inorganic fillers (bioactive glass, hydroxyapatite, amorphous calcium phosphate, graphene oxide, calcium chloride, zinc chloride, silica, and niobium pentoxide) in the literature that have been used to improve the different properties of DAs. These improvements can be seen in the form of improved hardness, higher modulus of elasticity, enhanced bond, flexural, and ultimate tensile strength, improved fracture toughness, reduced nanoleakage, remineralization of the adhesive–dentin interface, improved resin tag formation, greater radiopacity, antibacterial effect, and improved DC (observed for some fillers). Most of the studies dealing with the subject area are in vitro. Future in situ and in vivo studies are recommended to positively attest to the results of laboratory findings.

## 1. Introduction

**Figure 1:**
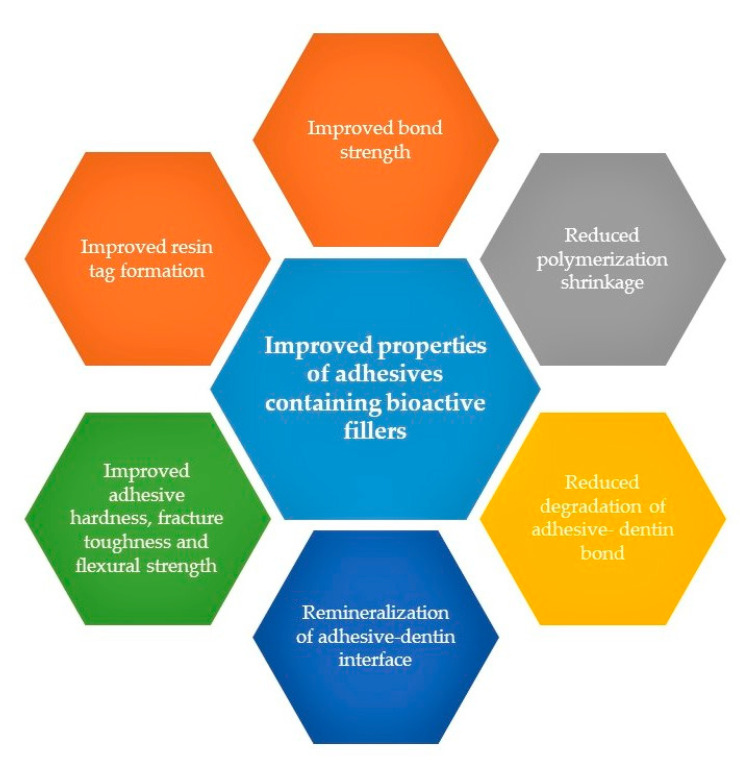
Improved properties shown by adhesives containing bioactive inoganic fillers [10,11].

Dental resin composites (DRCs) are polymeric materials used for restorative and aesthetic repairs [1]. They have gained a lot of popularity in recent years, and the number of composite restorations has surpassed 166 million in the United States (U.S.) only [2]. Monomers most commonly used in DRCs include bisphenol A-glycidyl methacrylate (Bis-GMA), Bis-GMA’s ethoxylated version (BisEMA), Triethylene glycol dimethacrylate (TEGDMA), and urethane dimethacrylate (UDMA) [3]. With the help of photo-initiators such as camphorquinone (CQ) with 2-dimethylamino ethyl methacrylate (DMAEMA) or ethyl-4-dimethylaminobenzoate (EDMAB), the resin matrix gets polymerized when it is exposed to a visible blue light source, such as light-emitting diodes (LEDs) [4]. Despite several advantageous characteristics of DRCs, they have questionable longevity (possessing a mean replacement time of 5.7 years) [5]. The most common reasons for the failure of DRCs include polymerization shrinkage, microleakage, and consequent secondary caries development [6]. Since their introduction in the market six decades ago, various modifications have been made in the composition of their polymer matrix and adhesive component in order to improve their longevity [1]. Dentin adhesives (DAs) are crucial for the clinical success of DRCs, as adhesion forms an intimate bond between the adhesive and tooth’s hard tissue [7]. This bond is formed when the adhesive components infiltrate the micro-porosities (created by etching) in the tooth structure to develop resin tags [8]. These tags form a mechanical interlocking between the adhesive and the tooth structure [9]. Various studies have proposed that the advances in polymer chemistry and incorporation of bioactive filler particles have augmented the properties of DAs [10,11]. These properties that are improved by the incorporation of bioactive inorganic fillers in the adhesives are summarized and presented in Figure 1.

## 2. Methodology

**Figure 2:**
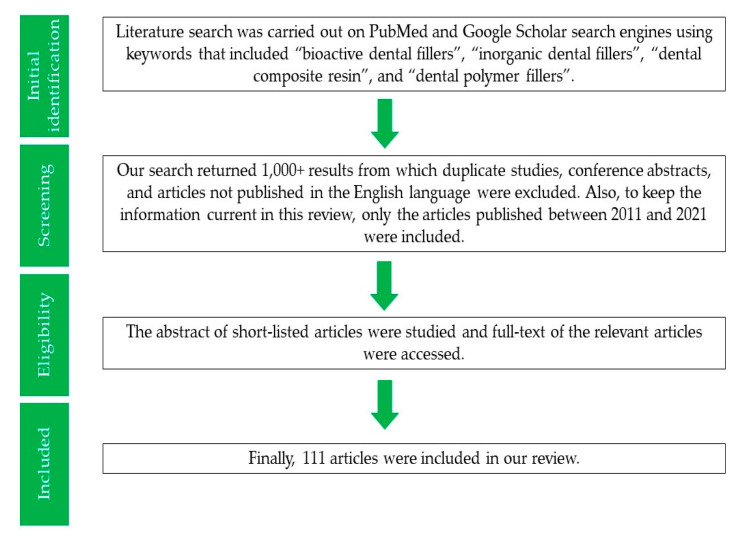
Flowchart depicting methodology adapted in our study.

The current review is aimed at summarizing information present in the literature regarding the improvement of the various properties of DAs after the addition of bioactive inorganic fillers. The methodology adapted for this review is presented in Figure 2.

## 3. Bioactive Inorganic Fillers

### 3.1. Bioactive Glass (BG) Fillers

#### 3.1.1. Overview

BGs are calcium-sodium phosphosilicate-based materials that can regenerate hard tissues [12]. The use of BGs in dentistry has recently increased due to their compositional similarity to the bone and dental hard tissues [13]. BGs are currently being used in dentistry for wide-ranging clinical applications and have been incorporated in toothpaste, glass-ionomer cement, and dental resin polymer composites [14,15,16]. The addition of BG fillers in the DAs can augment their properties, and this aspect will be discussed in detail below.

#### 3.1.2. Improvement of DA’s Properties

The inclusion of BG fillers in the adhesive could improve its hardness and modulus of elasticity [17]. In a previous study, Profeta et al. reported high micro-tensile bond strength (μTBS) and reduced nanoleakage at the resin–dentin interface when the adhesives were incorporated with BG doped fillers [18]. Sauro et al. echoed similar findings and reported an increase in the hardness and elastic modulus for BG-containing bonding systems instead of BG-free adhesive systems along with the dentin interface [19]. Yang et al. formerly reported that orthodontic adhesives containing BGs exhibited clinically acceptable shear bond strength (SBS) [20]. Biosilicate^®^ (BG-based material) has also been shown to increase the bond strength of the adhesives [21]. Jun et al. revealed that the addition of copper doped BG fillers in the adhesive resulted in the remineralization of the adhesive–dentin interface [22]. Niobophosphate BG fillers, when added to the commercial adhesives, have demonstrated greater radiopacity, hardness, and degree of conversion (DC), as compared to the adhesives devoid of BG fillers [23]. BG fillers could also reduce the marginal gap development by forming the apatite during the polymerization process [24]. The studies mentioned above provide evidence that DAs, after the inclusion of BG fillers, demonstrate enhanced properties. It should be noted that BG filler content could positively control the change in the adhesive’s properties; still, too high wt.% of the BG filler content could compromise the adhesive’s properties (especially the bond strength) [25].

#### 3.1.3. Mechanism of Improvement of DA’s Properties

Various mechanisms can be suggested that may lead to an improvement of DA’s properties when the BG fillers are incorporated in them. The inclusion of BG filler particles could cause a release of ions such as calcium and phosphate, which can cause remineralization of the adhesive–dentin interface [26]. Another similar reason has been advocated by various studies that have reported that the inclusion of BG-containing adhesive inhibits the bond degradation by protecting the collagen fibers via apatite formation [17,18]. The addition of BG fillers in the DAs could also cause an increase in the pH [27], resulting in remineralization of the adhesive–dentin interface. The presence of hydrated silica in the glass ensures that nucleation sites are present for the hydroxyapatite (HA) layer, hence promoting precipitation instantly after the dissolution initiates. This property helps to form an HA-like layer on the restoration surface that fills the marginal gap, thus hindering bacterial penetration and secondary caries development [28].

### 3.2. HA Fillers

#### 3.2.1. Overview

HA is part of the natural composition of human bones and teeth [29]. In the teeth, it is an essential constituent of enamel, dentin, and cementum tissues [29]. HA particles are remineralizing and promote natural apatite formation in human skeletal and dental tissues [30]. HA particles are small but decreasing their size from a micrometer to nanometer scale, increases their surface area available for reactivity [30]. Nano-HA particles are used for multiple applications in dentistry, including as a coating material for dental implants, as alveolar bone grafting material, inside a toothpaste to treat dentinal hypersensitivity, and as fillers in DAs [31,32]. The inclusion of HA particles can augment the properties of DAs, and multiple mechanisms could be possibly involved. The impact of their inclusion is discussed below.

#### 3.2.2. Improvement of DA’s Properties

**Figure 3:**
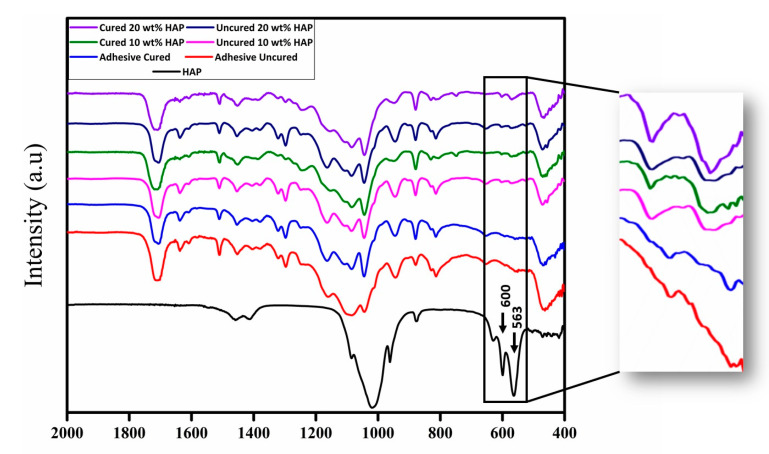
FTIR spectra of the HA nanoparticles, unmodified and modified EAs having low (10 wt.%) and high (20 wt.%) percentage of HA particles. The characteristic peaks at 563 and 600 cm^−1^ show evidence of the presence of HA in the uncured and cured dentin adhesive. Reprinted with permission from Al-Hamdan et al. [33]. Copyright 2020 MDPI.

**Figure 4:**
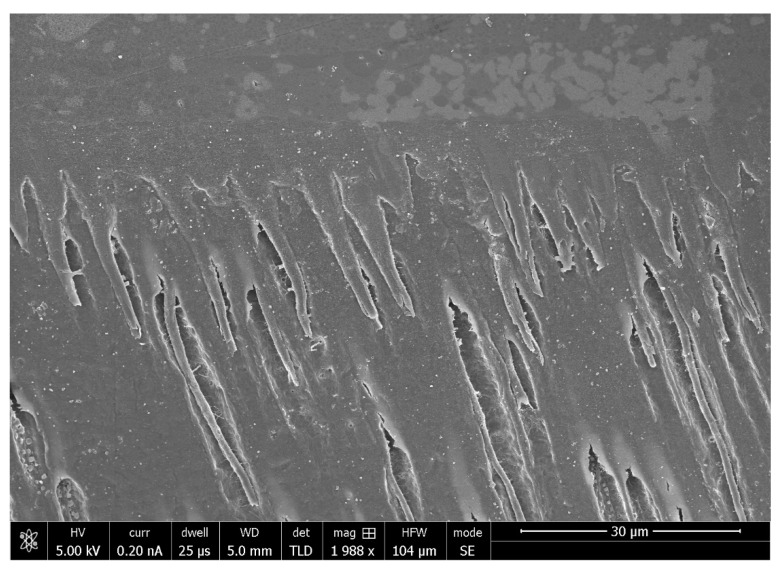
SEM micrograph of HA-containing adhesive demonstrating resin tag formation of varying depths. Reprinted with permission from Al-Hamdan et al. [33]. Copyright 2020 MDPI.

**Figure 5:**
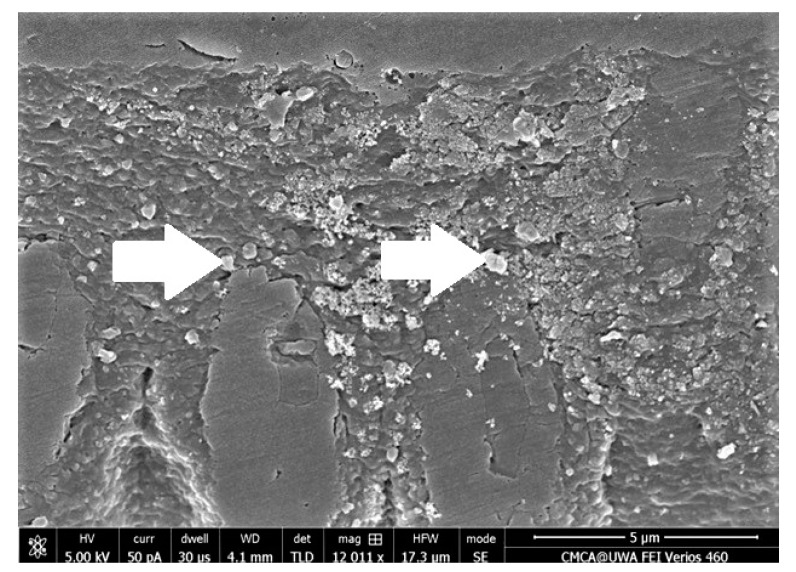
SEM micrograph of the hybrid layer showing widely distributed opaque HA nanoparticles (arrows). Reprinted with permission from Al-Hamdan et al. [33]. Copyright 2020 MDPI.

From the literature, it is evident that the inclusion of HA fillers in DAs improves their properties. Al-Hamdan et al. incorporated HA nanoparticles as fillers in their experimental adhesive (EA), which resulted in an improved bond strength being observed for HA-containing EA, as compared with the controls [33]. Additionally, the formation of typical apatite peaks demonstrating the presence of HA in the adhesive were noticed on Fourier Transform-Infrared (FTIR) spectra [33] (Figure 3, adapted from Al-Hamdan et al. [33]). In the same study, resin tags with varying depths were also seen depicting appropriate dentin interaction of the EA containing HA [33]. The resin tags formed by the HA-containing adhesives are shown in Figure 4, while their dispersion in the hybrid layer is shown in Figure 5 (adapted from Al-Hamdan et al.) [33]. Another previous study reported similar findings, and the inclusion of HA nanoparticles improved the μTBS of the adhesive and demonstrated proper resin tag formation on scanning electron microscopy (SEM) [34]. Various other studies have also reported an improved bond strength and surface micro-hardness of the adhesive after the addition of HA [33,34,35]. Though HA particles can improve the bond strength and resin tag formation ability of the DAs, a compromised DC has been reported by various studies with an increasing HA particle concentration [33,34]. Therefore, the addition of a high concentration of HA (>10 wt.%) should be made cautiously as it could lead to agglomeration of particles and increased viscosity, compromising the DC [36].

#### 3.2.3. Mechanism of Improvement of DA’s Properties

**Figure 6:**
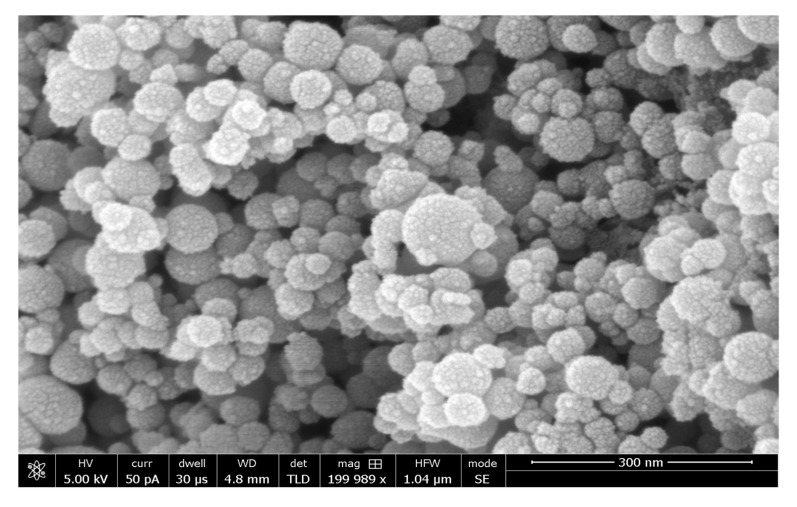
SEM micrograph of HA nanoparticles demonstrating their spherical shape and nano-size (<100 nm). Reprinted with permission from Al-Hamdan et al. [33]. Copyright 2020 MDPI.

A major reason for the improvement of DA’s properties is the remineralizing capabilities of HA particles owing to the presence of calcium and phosphate ions [37]. Another plausible reason could be the smaller size of HA particles. These particles are spherical and nano-sized as shown in Figure 6 (adapted from Al-Hamdan et al.) [33], thus ensuring adequate mineral release and availability of more adhesion area [38]. The HA particles are also able to biomineralize with the collagen fibers of the dental tissues [39], resulting in a stronger bond between the adhesive–dentin interface, demonstrated by improved bond strength. The improved micro-hardness illustrated by HA-containing adhesives could be explained by the presence of functionalized silane groups on the nanospheres of HA, which lower water sorption and consequently improve micro-hardness [34].

### 3.3. Amorphous Calcium Phosphate (ACP) Fillers

#### 3.3.1. Overview

ACP can be considered as a direct precursor of HA, which is directly involved in the remineralization of human bones and teeth [40]. ACP-based materials have various applications in dentistry, mainly related to remineralization of the tooth structure [41]. ACP is an unstable material that, upon exposure to water, changes into HA, releasing calcium and phosphate remineralizing ions [42]. This feature forms the basis of ACP’s bioactivity and can be exploited to promote remineralization via DAs.

#### 3.3.2. Improvement of DA’s Properties

The addition of ACP particles in DAs could prove to be helpful in reinforcing its properties. In several former studies, it has been demonstrated that the addition of up to 40 wt.% ACP particles in the adhesive did not have a negative impact on the dentin bond strength [38,43]. Melo et al. previously demonstrated that ACP-containing adhesives could penetrate dentinal tubules forming stable resin tags [38]. ACP fillers could fill the micro-gaps between the tooth-restoration surfaces, yielding a resilient bond [44]. There is also evidence of unimpaired DC and diminished volumetric polymerization shrinkage being observed with the addition of ACP fillers [45,46]. Additionally, the inclusion of ACP fillers does not considerably affect the curing light’s penetrating power [46].

#### 3.3.3. Mechanism of Improvement of DA’s Properties

Similar to HA and BG, the improvement in the DA’s properties could be attributed mainly to ACP fillers’ remineralizing capabilities. DAs with ACP particles release higher amounts of calcium and phosphate ions [38,43]. This release creates a supersaturated reservoir of ions which inhibits demineralization and promotes remineralization [44]. Additionally, these ions are deposited onto the tooth surface as apatitic minerals, similar to natural HA present in the teeth [47]. ACP filler particles could also neutralize acids by increasing calcium and phosphate ion discharge [48]. As ACP lacks the normal crystalline order and is highly soluble, it can quickly form apatite and increase the acidic solution’s pH that demineralizes the tooth structure [49]. ACP’s aggregation and excess water sorption is a concern, but the incorporation of mechanically milled ACP nanoparticles could improve homogenous filler dispersion and improved biaxial flexural strength [47].

### 3.4. Graphene Oxide (GO) Fillers

#### 3.4.1. Overview

Graphene-based materials (GBMs) are thermally and chemically stable and retain a high surface area [50]. GO is a nanomaterial that can be obtained by oxidizing graphite [51], but unlike other GBMs, it is hydrophilic due to the presence of oxygen in the functional groups [52]. GO is currently being used for various dental applications, including alveolar bone regeneration, treatment of oral cancer, drug delivery, as a biomaterial, and as a filler in DAs [51,53].

#### 3.4.2. Improvement of DA’s Properties

Various researchers have tested the use of GO as a filler to reinforce the properties of DAs. In an earlier study by Bregnocchi et al., GO-containing adhesive demonstrated comparable μTBS values to the controls [53]. AlFawaz et al. also incorporated GO particles in their EA and reported appropriate resin tag formation and greater μTBS values as compared to the adhesive without GO nanoparticles [54]. It was also reported in their study that the addition of 2 wt.% GO particles improved the μTBS of the adhesive more than that observed with the addition of 0.5 wt.% GO nanoparticles [54]. Bin-Shuwaish et al. also presented similar findings and revealed an improved μTBS paralleled with controls [11]. GO particles could also enhance the commercial primers’ bond strength, as reported by Khan et al. previously [55]. However, it should be noted that although an increase in GO content could increase the bond strength of the adhesive, a compromised DC could also occur, as demonstrated by several former studies [11,54]. As the DC is directly reliant on the percentage of the filler content [56], GO nanoparticles should be added cautiously in the adhesives.

#### 3.4.3. Mechanism of Improvement of DA’s Properties

**Figure 7:**
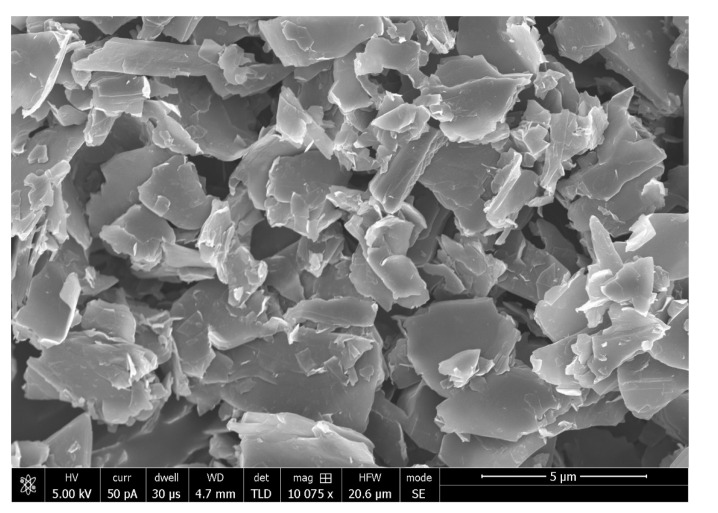
SEM micrograph demonstrating flake-shaped GO particles. Reprinted with permission from Al-Fawaz et al. [54]. Copyright 2020 MDPI.

The positive impact on the properties observed for the addition of GO fillers in the DAs could be attributed to their hydrophilic nature that could attract calcium ions to form HA, thus promoting remineralization of the adhesive–dentin interface [57]. The hydrophilic property of GO also improves the flow of the material, influencing resin tag formation and supporting the hybrid layer [11]. The oxidation of graphene to form GO results in the formation of flake-shaped sheets [54]. This sheet-like structure of GO particles could also reinforce the strength of the material [57]. The flake-shaped GO particles are shown in Figure 7 (adapted from Al-Fawaz et al.) [54].

### 3.5. Calcium Fluoride (CaF_2_) Fillers

#### 3.5.1. Overview

Demineralization of teeth can have deleterious effects on the health of dental tissues and overall oral hygiene [58]. Calcium and fluoride are considered essential ions that can remineralize tooth structure [58]. The inclusion of CaF_2_ nanoparticles in the material makes it antibacterial [59], stimulates fluoride release [60], and protects tooth structure against acidic attacks [61]. Historically, CaF_2_ nanoparticles have been added in different dental materials, including pit and fissure sealants [62], glass ionomer cements [63], and DAs [64] and have yielded positive results. The impact of CaF_2_ particles in DAs can enhance their various properties, which is a characteristic that is highlighted further below.

#### 3.5.2. Improvement of DA’s Properties

Studies from the last ten years demonstrating the effect of the addition of CaF_2_ fillers on the properties of DAs are scarce. CaF_2_ nanoparticles reinforce many properties of DAs, but they are largely based on their remineralization capabilities and antibacterial potential. Essam et al. previously incorporated CaF_2_ nanoparticles in two-step self-etch adhesives in an in vivo study and reported that due to the presence of these filler particles, remineralization of the caries-affected dentin was noticed [65]. The incorporation of CaF_2_ nanoparticles possessing high surface area in dental composites results in overall high levels of fluoride and calcium ions, even at low filler concentration [66]. Another earlier study reported that a 2.0 wt.% CaF_2_ filler containing composite demonstrated the highest Vickers hardness, compared with the controls [67]. In a previous study, nanocomposites containing CaF_2_ revealed strong antibacterial and ion releasing properties [61]. A significant concern with the use of CaF_2_ nanoparticles is their stability; however, they are aptly stable in the oral environment than generally presumed [68].

#### 3.5.3. Mechanism of Improvement of DA’s Properties

It is a well-known fact that the presence of fluoride promotes the precipitation of calcium and phosphate ions to form fluorapatite, which is more chemically stable and resistant to carious attacks [69]. CaF_2_ nanoparticles serve as a reservoir of fluoride that could be used for the remineralization of tooth structure [70]. Considering secondary caries as one of the problems affecting the longevity of the DRC restorations, the incorporation of fluoride-releasing CaF_2_ nanoparticles ensures a sustained release of fluoride resulting in an overall anti-caries effect [71].

### 3.6. Zn Chloride (ZnCl_2_) Fillers

#### 3.6.1. Overview

Zn has been incorporated in DAs to achieve reduced collagen degradation, promote remineralization of the resin–dentin interface, and to sustain bonding efficacy [72,73]. Numerous Zn salts, including ZnO, ZnN_3_, Zn-methacrylate, and ZnCl_2,_ have been added previously in the DAs [74,75,76]. Amongst these salts, ZnCl_2_ presents a faster dissolution rate and can result in greater saturation of Zn^2+^ in the DAs [77]. The impact of ZnCl_2_ addition on the various properties of the DAs will now be discussed below.

#### 3.6.2. Improvement of DA’s Properties

Zn-doped DAs can be attained by adding 2 wt.% ZnCl_2_ particles in the dental adhesives [78]. In addition, this concentration of ZnCl_2_ does not affect DAs translucency, thus ensuring that adequate polymerization of monomers is achieved [77]. A former study validated that ZnCl_2_ doped DA led to a reduction in the nanoleakage and better sealability at the dentin interface [79]. In another study, ZnCl_2_-containing adhesive presented higher flexural strength compared with the controls, and the DC was also not affected [80]. Campos et al. performed a study and reported that the bond strength of ZnCl_2_-containing adhesive did not differ from the control group adhesive [81]. A study by Navarra et al. reported that ZnCl_2_ adhesives demonstrated DC values [77] that were comparable to DC values shown by commercial DAs in another study [82]. ZnCl_2_ is a highly soluble salt, and higher amounts of leaching could occur in the oral environment, thus affecting properties of the material over long periods of time [77].

#### 3.6.3. Mechanism of Improvement of DA’s Properties

One of the reasons for the closeness of DC observed for ZnCl_2_-containing adhesives and commercial adhesives is the refractive index (RI) of ZnCl_2_ (1.68), which is closer to the RI of the organic matrix (1.5) [77]. A high DC ensures that an adequate number of monomers are photo-polymerized, prolonging the longevity of the DRC restoration [83]. Another mechanism by which degradation of the bond is prevented could be the hybridization of demineralized dentin that reduces collagen degradation, which was observed for ZnCl_2_ adhesives in a previous study [84]. Biomineralization of dental hard tissues initiated by adhesives containing Zn salts is also one plausible reason for the improved mineralization of the interface, crystallinity, and repair of demineralized dentin tissue, which was observed in several former studies [76,85,86].

### 3.7. Silica Fillers

#### 3.7.1. Overview

Silica particles are used in different materials related to dentistry. Most noticeably, they are used in cements (calcium silicate and glass ionomer) [30,87], toothpastes [88], dental ceramics [89], and in DAs [90]. The improvement of DA properties is well-documented in the literature, an aspect discussed below in detail.

#### 3.7.2. Improvement of DA’s Properties

Improved µTBS of adhesives are noted after the incorporation of silica particles [91]. Guo et al. reported that a mixture of zirconia and silica could enhance fracture toughness and flexural strength of DRCs [92]. In one study, it was demonstrated that Zn doped silica nanoparticle fillers could improve the antibacterial and flexural strength and modulus, hardness, and compressive strength of DRCs [93]. An earlier study also reported that the addition of sepiolite (nanoparticles based on phyllosilicates) in the adhesive increases its bond strength [94]. Timpe et al. also reported that the addition of nano-sized silica filler particles resulted in their uniform dispersion without any marked effect on the viscosity of the DRCs [95]. Hence, the use of silica nano-sized particles in the adhesive is useful, while larger-sized silica particles could compromise its properties.

#### 3.7.3. Mechanism of Improvement of DA’s Properties

The silica particles could enhance the properties of the adhesive by remineralizing the adhesive–dentin interface, as they encourage the formation of calcium-phosphate precursors and act as a nucleating mineral [96]. The occurrence of silica particles in the material also ensures that calcium particles are attracted to develop a stable calcium silicate bioactive compound that can attach to phosphorus [97]. Resin tag formation is enhanced by the presence of silica particles [91]. The probable reason for this could be that silica nanoparticles are spherical-shaped, and this morphology helps the material to disperse and flow properly without any significant effect on the viscosity [98].

### 3.8. Niobium Pentoxide (Nb_2_O_5_) Fillers

#### 3.8.1. Overview

Niobium is a metal that is used in metallurgy to augment the properties of different metals [99]. The use of niobium pentoxide (Nb_2_O_5_) related to dental applications has risen recently. It can promote HA-like crystal growth when it comes in contact with saliva and could be used to yield endodontic sealers with enhanced radiopacity and micro-hardness [100]. Additionally, Nb_2_O_5_ could also be used as a filler in DAs to augment their properties [101].

#### 3.8.2. Improvement of DA’s Properties

A previous study has shown that the addition of Nb_2_O_5_ in DAs improves its micro-hardness, radiopacity, and polymerization rate [101]. In the same study [101], the Nb_2_O_5_ containing DA was able to penetrate through the hybrid layer giving stability to the material. In another study, the addition of 2 wt.% silica and niobium particles led to an increase in the mineral deposition and improved bond strength [102]. Garcia et al. reported in their study that Nb_2_O_5_-containing DA demonstrated increased opacity, and appropriate ultimate tensile strength [103].

#### 3.8.3. Mechanism of Improvement of DA’s Properties

The improved micro-hardness and radiopacity of Nb_2_O_5_ containing DAs are owed to the inorganic nature of Nb_2_O_5_ particles. The addition of these hard particles in the soft resin matrix improves its micro-hardness and radiopacity [101]. The incorporation of Nb_2_O_5_ particles in the polymer matrix increases the reactivity of the system and decreases the required energy to yield free radicals, thus causing an increased polymerization rate [101]. In addition, the infiltration of Nb_2_O_5_ particles into a collagen matrix that is uncovered by the etching of acid could encourage the formation of a hybrid layer that is less degradable with improved biological properties [101].

### 3.9. Other Bioactive Inorganic Fillers

Certain other fillers that have been added to DAs, along with the specific properties that they improve in the DAs, are summarized in Table 1.

## 4. Conclusions

This review concludes that the addition of various bioactive inorganic fillers can improve multiple properties of DAs. These improvements can be seen in the form of improved hardness, higher modulus of elasticity, enhanced bond, flexural, and ultimate tensile strength, improved fracture toughness, reduced nanoleakage, remineralization of adhesive–dentin interface, improved resin tag formation, greater radiopacity, and improved DC (observed for some fillers), and antibacterial effect. Most of the studies dealing with the subject area are in vitro. Future in situ and in vivo studies are recommended to attest to the results of laboratory findings.

## Figures and Tables

**Table 1:** Other bioactive inorganic fillers used to enhance properties of DAs mentioned in the literature.

S.No.	Bioactive Fillers	Improves Adhesive’s Properties	Reason(s) for the Improved Properties	Selected Reference(s)
1.	Silver (Ag) based fillers	√	Antibacterial property, remineralizing effect, high surface area	[104,105,106]
2.	Niobic acid (Nb_2_O_5_·*n* H_2_O)	√	Improved resistance against solvents, bioactive inorganic nature	[103]
3.	Chitosan	√	Antibacterial	[39]
4.	Zn based fillers	√	Interference with the matrix metalloproteinases (MMPs)-mediated collagen degradation, remineralizing effect due to slow Zn liberation resulting in ZnO rich layer	[75,107]
5.	Cerium dioxide (CeO_2_) filler	√	Improved radiopacity, sufficient dispersion in the DA	[108]
6.	Tantalum oxide (Ta_2_O_5_) filler	√	Improved radiopacity, improvement of attraction between polymer chains and solvent molecules (resulting in less degradation of adhesive-dentin bond)	[109]
7.	Zirconia (Zr) based fillers	√	Improved radiopacity and micro-hardness	[110]
8.	Quaternary ammonium salts (QAS)	√	Antibacterial effect	[111]

## References

[1] Pfeifer C.S. (2017). Polymer-Based Direct Filling Materials. Dent. Clin. N. Am..

[2] Stewart C.A., Hong J.H., Hatton B.D., Finer Y. (2018). Responsive antimicrobial dental adhesive based on drug-silica co-assembled particles. Acta Biomater..

[3] Gajewski V.E., Pfeifer C.S., Froes-Salgado N.R., Boaro L.C., Braga R.R. (2012). Monomers used in resin composites: Degree of conversion, mechanical properties and water sorption/solubility. Braz. Dent. J..

[4] Pratap B., Gupta R.K., Bhardwaj B., Nag M. (2019). Resin based restorative dental materials: Characteristics and future perspectives. Jpn. Dent. Sci. Rev..

[5] Bourbia M., Finer Y. (2018). Biochemical Stability and Interactions of Dental Resin Composites and Adhesives with Host and Bacteria in the Oral Cavity: A Review. J. Can. Dent. Assoc..

[6] Ferracane J.L. (2017). Models of Caries Formation around Dental Composite Restorations. J. Dent. Res..

[7] Manuja N., Nagpal R., Pandit I.K. (2012). Dental adhesion: Mechanism, techniques and durability. J. Clin. Pediatr. Dent..

[8] Sofan E., Sofan A., Palaia G., Tenore G., Romeo U., Migliau G. (2017). Classification review of dental adhesive systems: From the IV generation to the universal type. Ann. Stomatol..

[9] Spencer P., Jonggu Park Q.Y., Misra A., Bohaty B.S., Singh V., Parthasarathy R., Sene F., de Paiva Goncalves S.E., Laurence J. (2012). Durable bonds at the adhesive/dentin interface: An impossible mission or simply a moving target?. Braz. Dent. Sci..

[10] Chi M., Qi M., A L., Wang P., Weir M.D., Melo M.A., Sun X., Dong B., Li C., Wu J. (2019). Novel Bioactive and Therapeutic Dental Polymeric Materials to Inhibit Periodontal Pathogens and Biofilms. Int. J. Mol. Sci..

[11] Bin-Shuwaish M.S.M., Maawadh A.M., Al-Hamdan R.S., Alresayes S., Ali T., Almutairi B., Vohra F., Abduljabbar T. (2020). Influence of graphene oxide filler content on the dentin bond integrity, degree of conversion and bond strength of experimental adhesive. A SEM, micro-Raman, FTIR and microtensile study. Mater. Res. Express..

[12] Al-Harbi N., Mohammed H., Al-Hadeethi Y., Bakry A.S., Umar A., Hussein M.A., Abbassy M.A., Vaidya K.G., Berakdar G.A., Mkawi E.M. (2021). Silica-Based Bioactive Glasses and Their Applications in Hard Tissue Regeneration: A Review. Pharmaceuticals.

[13] Sawant K., Pawar A.M. (2020). Bioactive glass in dentistry: A systematic review. Saudi J. Oral Sci..

[14] Fernando D., Attik N., Pradelle-Plasse N., Jackson P., Grosgogeat B., Colon P. (2017). Bioactive glass for dentin remineralization: A systematic review. Mater. Sci. Eng. C Mater. Biol. Appl..

[15] Kim H.J., Bae H.E., Lee J.E., Park I.S., Kim H.J., Kwon J., Kim D.S. (2021). Effects of bioactive glass incorporation into glass ionomer cement on demineralized dentin. Sci. Rep..

[16] Jang J.H., Lee M.G., Ferracane J.L., Davis H., Bae H.E., Choi D., Kim D.S. (2018). Effect of bioactive glass-containing resin composite on dentin remineralization. J. Dent..

[17] Profeta A.C., Mannocci F., Foxton R.M., Thompson I., Watson T.F., Sauro S. (2012). Bioactive effects of a calcium/sodium phosphosilicate on the resin-dentine interface: A microtensile bond strength, scanning electron microscopy, and confocal microscopy study. Eur. J. Oral Sci..

[18] Profeta A.C., Mannocci F., Foxton R., Watson T.F., Feitosa V.P., De Carlo B., Mongiorgi R., Valdre G., Sauro S. (2013). Experimental etch-and-rinse adhesives doped with bioactive calcium silicate-based micro-fillers to generate therapeutic resin-dentin interfaces. Dent. Mater..

[19] Sauro S., Osorio R., Watson T.F., Toledano M. (2012). Therapeutic effects of novel resin bonding systems containing bioactive glasses on mineral-depleted areas within the bonded-dentine interface. J. Mater. Sci. Mater. Med..

[20] Yang S.Y., Kim S.H., Choi S.Y., Kim K.M. (2016). Acid Neutralizing Ability and Shear Bond Strength Using Orthodontic Adhesives Containing Three Different Types of Bioactive Glass. Materials.

[21] de Morais R.C., Silveira R.E., Chinelatti M., Geraldeli S., de Carvalho Panzeri Pires-de-Souza F. (2018). Bond strength of adhesive systems to sound and demineralized dentin treated with bioactive glass ceramic suspension. Clin. Oral Investig..

[22] Jun S.K., Yang S.A., Kim Y.J., El-Fiqi A., Mandakhbayar N., Kim D.S., Roh J., Sauro S., Kim H.W., Lee J.H. (2018). Multi-functional nano-adhesive releasing therapeutic ions for MMP-deactivation and remineralization. Sci. Rep..

[23] Carneiro K.K., Araujo T.P., Carvalho E.M., Meier M.M., Tanaka A., Carvalho C.N., Bauer J. (2018). Bioactivity and properties of an adhesive system functionalized with an experimental niobium-based glass. J. Mech. Behav. Biomed. Mater..

[24] Tiskaya M., Shahid S., Gillam D., Hill R. (2021). The use of bioactive glass (BAG) in dental composites: A critical review. Dent. Mater..

[25] Par M., Tarle Z., Hickel R., Ilie N. (2018). Dentin Bond Strength of Experimental Composites Containing Bioactive Glass: Changes During Aging for up to 1 Year. J. Adhes. Dent..

[26] Sauro S., Osorio R., Osorio E., Watson T.F., Toledano M. (2013). Novel light-curable materials containing experimental bioactive micro-fillers remineralise mineral-depleted bonded-dentine interfaces. J. Biomater. Sci. Polym. Ed..

[27] Par M., Attin T., Tarle Z., Tauböck T.T. (2020). A New Customized Bioactive Glass Filler to Functionalize Resin Composites: Acid-Neutralizing Capability, Degree of Conversion, and Apatite Precipitation. J. Clin. Med..

[28] Khvostenko D., Hilton T.J., Ferracane J.L., Mitchell J.C., Kruzic J.J. (2016). Bioactive glass fillers reduce bacterial penetration into marginal gaps for composite restorations. Dent. Mater..

[29] Pajor K., Pajchel L., Kolmas J. (2019). Hydroxyapatite and Fluorapatite in Conservative Dentistry and Oral Implantology-A Review. Materials.

[30] Moheet I.A., Luddin N., Rahman I.A., Kannan T.P., Nik Abd Ghani N.R., Masudi S.M. (2019). Modifications of Glass Ionomer Cement Powder by Addition of Recently Fabricated Nano-Fillers and Their Effect on the Properties: A Review. Eur. J. Dent..

[31] Bordea I.R., Candrea S., Alexescu G.T., Bran S., Baciut M., Baciut G., Lucaciu O., Dinu C.M., Todea D.A. (2020). Nano-hydroxyapatite use in dentistry: A systematic review. Drug Metab. Rev..

[32] Giannini M., Makishi P., Ayres A.P., Vermelho P.M., Fronza B.M., Nikaido T., Tagami J. (2015). Self-etch adhesive systems: A literature review. Braz. Dent. J..

[33] Al-Hamdan R.S., Almutairi B., Kattan H.F., Alsuwailem N.A., Farooq I., Vohra F., Abduljabbar T. (2020). Influence of Hydroxyapatite Nanospheres in Dentin Adhesive on the Dentin Bond Integrity and Degree of Conversion: A Scanning Electron Microscopy (SEM), Raman, Fourier Transform-Infrared (FTIR), and Microtensile Study. Polymers.

[34] Al-Hamdan R.S., Almuthairi B., Kattan H.F., Alresayes S., Abduljabbar T., Vohra F. (2020). Assessment of Hydroxyapatite Nanospheres Incorporated Dentin Adhesive. A SEM/EDX, Micro-Raman, Microtensile and Micro-Indentation Study. Coatings.

[35] Kavrik F., Kucukyilmaz E. (2019). The effect of different ratios of nano-sized hydroxyapatite fillers on the micro-tensile bond strength of an adhesive resin. Microsc. Res. Tech..

[36] Wagner A., Belli R., Stotzel C., Hilpert A., Muller F.A., Lohbauer U. (2013). Biomimetically- and hydrothermally-grown HAp nanoparticles as reinforcing fillers for dental adhesives. J. Adhes. Dent..

[37] Pepla E., Besharat L.K., Palaia G., Tenore G., Migliau G. (2014). Nano-hydroxyapatite and its applications in preventive, restorative and regenerative dentistry: A review of literature. Ann. Stomatol..

[38] Melo M.A., Cheng L., Zhang K., Weir M.D., Rodrigues L.K., Xu H.H. (2013). Novel dental adhesives containing nanoparticles of silver and amorphous calcium phosphate. Dent. Mater..

[39] Zhou W., Liu S., Zhou X., Hannig M., Rupf S., Feng J., Peng X., Cheng L. (2019). Modifying Adhesive Materials to Improve the Longevity of Resinous Restorations. Int. J. Mol. Sci..

[40] Hou A., Luo J., Zhang M., Li J., Chu W., Liang K., Yang J., Li J. (2020). Two-in-one strategy: A remineralizing and anti-adhesive coating against demineralized enamel. Int. J. Oral Sci..

[41] Gupta N., Mohan Marya C., Nagpal R., Singh Oberoi S., Dhingra C. (2016). A Review of Casein Phosphopeptide-Amorphous Calcium Phosphate (CPP-ACP) and Enamel Remineralization. Compend. Contin. Educ. Dent..

[42] Iafisco M., Degli Esposti L., Ramirez-Rodriguez G.B., Carella F., Gomez-Morales J., Ionescu A.C., Brambilla E., Tampieri A., Delgado-Lopez J.M. (2018). Fluoride-doped amorphous calcium phosphate nanoparticles as a promising biomimetic material for dental remineralization. Sci. Rep..

[43] Chen C., Weir M.D., Cheng L., Lin N.J., Lin-Gibson S., Chow L.C., Zhou X., Xu H.H. (2014). Antibacterial activity and ion release of bonding agent containing amorphous calcium phosphate nanoparticles. Dent. Mater..

[44] Zhang L., Weir M.D., Chow L.C., Reynolds M.A., Xu H.H. (2016). Rechargeable calcium phosphate orthodontic cement with sustained ion release and re-release. Sci. Rep..

[45] Marovic D., Sariri K., Demoli N., Ristic M., Hiller K.A., Skrtic D., Rosentritt M., Schmalz G., Tarle Z. (2016). Remineralizing amorphous calcium phosphate based composite resins: The influence of inert fillers on monomer conversion, polymerization shrinkage, and microhardness. Croat. Med. J..

[46] Par M., Marovic D., Skenderovic H., Gamulin O., Klaric E., Tarle Z. (2017). Light transmittance and polymerization kinetics of amorphous calcium phosphate composites. Clin. Oral Investig..

[47] Zhao J., Liu Y., Sun W.B., Zhang H. (2011). Amorphous calcium phosphate and its application in dentistry. Chem. Cent. J..

[48] Moreau J.L., Sun L., Chow L.C., Xu H.H. (2011). Mechanical and acid neutralizing properties and bacteria inhibition of amorphous calcium phosphate dental nanocomposite. J. Biomed. Mater. Res. B Appl. Biomater..

[49] Balhaddad A.A., Kansara A.A., Hidan D., Weir M.D., Xu H.H.K., Melo M.A.S. (2019). Toward dental caries: Exploring nanoparticle-based platforms and calcium phosphate compounds for dental restorative materials. Bioact. Mater..

[50] Baig M.S., Fleming G.J. (2015). Conventional glass-ionomer materials: A review of the developments in glass powder, polyacid liquid and the strategies of reinforcement. J. Dent..

[51] Ge Z., Yang L., Xiao F., Wu Y., Yu T., Chen J., Lin J., Zhang Y. (2018). Graphene Family Nanomaterials: Properties and Potential Applications in Dentistry. Int. J. Biomater..

[52] Wei N., Lv C., Xu Z. (2014). Wetting of graphene oxide: A molecular dynamics study. Langmuir.

[53] Bregnocchi A., Zanni E., Uccelletti D., Marra F., Cavallini D., De Angelis F., De Bellis G., Bossu M., Ierardo G., Polimeni A. (2017). Graphene-based dental adhesive with anti-biofilm activity. J. Nanobiotechnol..

[54] AlFawaz Y.F., Almutairi B., Kattan H.F., Zafar M.S., Farooq I., Naseem M., Vohra F., Abduljabbar T. (2020). Dentin Bond Integrity of Hydroxyapatite Containing Resin Adhesive Enhanced with Graphene Oxide Nano-Particles—An SEM, EDX, Micro-Raman, and Microtensile Bond Strength Study. Polymers.

[55] Khan A.A., Al-Khureif A.A., Saadaldin S.A., Mohamed B.A., Musaibah A.S.O., Divakar D.D., Eldwakhly E. (2019). Graphene oxide-based experimental silane primers enhance shear bond strength between resin composite and zirconia. Eur. J. Oral Sci..

[56] Aguiar T.R., de Oliveira M., Arrais C.A., Ambrosano G.M., Rueggeberg F., Giannini M. (2015). The effect of photopolymerization on the degree of conversion, polymerization kinetic, biaxial flexure strength, and modulus of self-adhesive resin cements. J. Prosthet. Dent..

[57] Alshahrani A., Bin-Shuwaish M.S., Al-Hamdan R.S., Almohareb T., Maawadh A.M., Al Deeb M., Alhenaki A.M., Abduljabbar T., Vohra F. (2020). Graphene oxide nano-filler based experimental dentine adhesive. A SEM/EDX, Micro-Raman and microtensile bond strength analysis. J. Appl. Biomater. Funct. Mater..

[58] Dai Z., Liu M., Ma Y., Cao L., Xu H.H.K., Zhang K., Bai Y. (2019). Effects of Fluoride and Calcium Phosphate Materials on Remineralization of Mild and Severe White Spot Lesions. Biomed Res. Int..

[59] Kulshrestha S., Khan S., Hasan S., Khan M.E., Misba L., Khan A.U. (2016). Calcium fluoride nanoparticles induced suppression of Streptococcus mutans biofilm: An in vitro and in vivo approach. Appl. Microbiol. Biotechnol..

[60] Cheng L., Weir M.D., Xu H.H., Kraigsley A.M., Lin N.J., Lin-Gibson S., Zhou X. (2012). Antibacterial and physical properties of calcium-phosphate and calcium-fluoride nanocomposites with chlorhexidine. Dent. Mater..

[61] Mitwalli H., Balhaddad A.A., AlSahafi R., Oates T.W., Melo M.A.S., Xu H.H.K., Weir M.D. (2020). Novel CaF2 Nanocomposites with Antibacterial Function and Fluoride and Calcium Ion Release to Inhibit Oral Biofilm and Protect Teeth. J. Funct. Biomater..

[62] Fei X., Li Y., Weir M.D., Baras B.H., Wang H., Wang S., Sun J., Melo M.A.S., Ruan J., Xu H.H.K. (2020). Novel pit and fissure sealant containing nano-CaF2 and dimethylaminohexadecyl methacrylate with double benefits of fluoride release and antibacterial function. Dent. Mater..

[63] Yi J., Dai Q., Weir M.D., Melo M.A.S., Lynch C.D., Oates T.W., Zhang K., Zhao Z., Xu H.H.K. (2019). A nano-CaF2-containing orthodontic cement with antibacterial and remineralization capabilities to combat enamel white spot lesions. J. Dent..

[64] Mahrous A., Radwan M.M., Kamel S.M. (2020). Micro-Shear Bond Strength of Novel MDP Calcium-Fluoride-Releasing Self-Adhesive Resin Cement After Thermocycling. Int. J. Periodontics Restor. Dent..

[65] Essam M.N., Niazy M.A., Farouk H., Mostafa A.A. (2019). The Remineralizing Effect of Incorporating Ca-Phosphate and Ca-Fluoride Nanoparticles into the Self-Etch Adhesives Used in Restoring Class I Cavities. Al-Azhar Dent. J. Girls.

[66] Weir M.D., Moreau J.L., Levine E.D., Strassler H.E., Chow L.C., Xu H.H. (2012). Nanocomposite containing CaF(2) nanoparticles: Thermal cycling, wear and long-term water-aging. Dent. Mater..

[67] Lukomska-Szymanska M., Kleczewska J., Nowak J., Prylinski M., Szczesio A., Podlewska M., Sokolowski J., Lapinska B. (2016). Mechanical Properties of Calcium Fluoride-Based Composite Materials. Biomed Res. Int..

[68] Firoozmand L.M., Noleto L.E., Gomes I.A., Bauer J.R., Ferreira M.C. (2015). Effect of Fluoride and Simplified Adhesive Systems on the Bond Strength of Primary Molars and Incisors. Braz. Dent. J..

[69] Rosin-Grget K., Peros K., Sutej I., Basic K. (2013). The cariostatic mechanisms of fluoride. Acta Med. Acad..

[70] Koeser J., Carvalho T.S., Pieles U., Lussi A. (2014). Preparation and optimization of calcium fluoride particles for dental applications. J. Mater. Sci. Mater. Med..

[71] Abou Neel E.A., Bozec L., Perez R.A., Kim H.W., Knowles J.C. (2015). Nanotechnology in dentistry: Prevention, diagnosis, and therapy. Int. J. Nanomed..

[72] Toledano M., Yamauti M., Ruiz-Requena M.E., Osorio R. (2012). A ZnO-doped adhesive reduced collagen degradation favouring dentine remineralization. J. Dent..

[73] Toledano M., Sauro S., Cabello I., Watson T., Osorio R. (2013). A Zn-doped etch-and-rinse adhesive may improve the mechanical properties and the integrity at the bonded-dentin interface. Dent. Mater..

[74] Barcellos D.C., Fonseca B.M., Pucci C.R., Cavalcanti B., Persici Ede S., Goncalves S.E. (2016). Zn-doped etch-and-rinse model dentin adhesives: Dentin bond integrity, biocompatibility, and properties. Dent. Mater..

[75] Feitosa V.P., Pomacondor-Hernandez C., Ogliari F.A., Leal F., Correr A.B., Sauro S. (2014). Chemical interaction of 10-MDP (methacryloyloxi-decyl-dihydrogen-phosphate) in zinc-doped self-etch adhesives. J. Dent..

[76] Toledano M., Aguilera F.S., Osorio E., Cabello I., Toledano-Osorio M., Osorio R. (2015). Bond strength and bioactivity of Zn-doped dental adhesives promoted by load cycling. Microsc. Microanal..

[77] Almeida G.S., da Silva E.M., Guimaraes J.G.A., da Silva R.N.L., Dos Santos G.B., Poskus L.T. (2017). ZnCl2 Incorporated into Experimental Adhesives: Selected Physicochemical Properties and Resin-Dentin Bonding Stability. Biomed Res. Int..

[78] Pomacondor-Hernandez C., Osorio R., Aguilera F.S., Cabello I., De Goes M., Toledano M. (2015). Effect of zinc-doping in physicochemical properties of dental adhesives. Am. J. Dent..

[79] Toledano M., Osorio R., Osorio E., Cabello I., Toledano-Osorio M., Aguilera F.S. (2017). A zinc chloride-doped adhesive facilitates sealing at the dentin interface: A confocal laser microscopy study. J. Mech. Behav. Biomed. Mater..

[80] Oliveira C.A., Campos R.M., Macedo J.P., Silva A.R., Maximo L.N., Silva T.M., Franca F.M., Turssi C.P., Basting R.T., Goncalves S.E.P. (2019). Incorporation of ZnCl2 into an etch-and-rinse adhesive system on flexural strength, degree of conversion and bond durability to caries-affected dentin. Am. J. Dent..

[81] Campos R.M.P., Oliveira C.A.R., Macedoa J.P.C., Françaa F.M.G., Basting R.T., Turssia C.P., Silva T.M., Gonçalves S.E.P., Amaral F.L.B. (2019). Effect of zinc chloride added to self-etching primer on bond strength to caries-affected dentin and chemical-physical-mechanical properties of adhesives. Int. J. Adhes. Adhes..

[82] Navarra C.O., Breschi L., Turco G., Diolosa M., Fontanive L., Manzoli L., Di Lenarda R., Cadenaro M. (2012). Degree of conversion of two-step etch-and-rinse adhesives: In situ micro-Raman analysis. J. Dent..

[83] AlShaafi M.M. (2017). Factors affecting polymerization of resin-based composites: A literature review. Saudi Dent. J..

[84] Osorio R., Yamauti M., Osorio E., Ruiz-Requena M.E., Pashley D.H., Tay F.R., Toledano M. (2011). Zinc reduces collagen degradation in demineralized human dentin explants. J. Dent..

[85] Toledano M., Aguilera F.S., Osorio E., Cabello I., Toledano-Osorio M., Osorio R. (2015). Self-etching zinc-doped adhesives improve the potential of caries-affected dentin to be functionally remineralized. Biointerphases.

[86] Osorio R., Osorio E., Cabello I., Toledano M. (2014). Zinc induces apatite and scholzite formation during dentin remineralization. Caries Res..

[87] Duarte M.A.H., Marciano M.A., Vivan R.R., Tanomaru Filho M., Tanomaru J.M.G., Camilleri J. (2018). Tricalcium silicate-based cements: Properties and modifications. Braz. Oral Res..

[88] Philpotts C.J., Cariddi E., Spradbery P.S., Joiner A. (2017). In vitro evaluation of a silica whitening toothpaste containing blue covarine on the colour of teeth containing anterior restoration materials. J. Dent..

[89] Monteiro P., Brito P., Pereira J., Alves R. (2012). The importance of the optical properties in dental silica-based ceramics. J. Calif. Dent. Assoc..

[90] Yan H., Wang S., Han L., Peng W., Yi L., Guo R., Liu S., Yang H., Huang C. (2018). Chlorhexidine-encapsulated mesoporous silica-modified dentin adhesive. J. Dent..

[91] Alhenaki A.M., Attar E.A., Alshahrani A., Farooq I., Vohra F., Abduljabbar T. (2021). Dentin Bond Integrity of Filled and Unfilled Resin Adhesive Enhanced with Silica Nanoparticles—An SEM, EDX, Micro-Raman, FTIR and Micro-Tensile Bond Strength Study. Polymers.

[92] Guo G., Fan Y., Zhang J.F., Hagan J.L., Xu X. (2012). Novel dental composites reinforced with zirconia-silica ceramic nanofibers. Dent. Mater..

[93] Bai X., Lin C., Wang Y., Ma J., Wang X., Yao X., Tang B. (2020). Preparation of Zn doped mesoporous silica nanoparticles (Zn-MSNs) for the improvement of mechanical and antibacterial properties of dental resin composites. Dent. Mater..

[94] Fallahzadeh F., Safarzadeh-Khosroshahi S., Atai M. (2017). Dentin bonding agent with improved bond strength to dentin through incorporation of sepiolite nanoparticles. J. Clin. Exp. Dent..

[95] Timpe N., Fullriede H., Borchers L., Stiesch M., Behrens P., Menzel H. (2014). Nanoporous silica nanoparticles with spherical and anisotropic shape as fillers in dental composite materials. BioNanoMaterials.

[96] Watson T.F., Atmeh A.R., Sajini S., Cook R.J., Festy F. (2014). Present and future of glass-ionomers and calcium-silicate cements as bioactive materials in dentistry: Biophotonics-based interfacial analyses in health and disease. Dent. Mater..

[97] Profeta A.C. (2014). Dentine bonding agents comprising calcium-silicates to support proactive dental care: Origins, development and future. Dent. Mater. J..

[98] Rodríguez H.A., Casanova H. (2018). Effects of silica nanoparticles and silica-zirconia nanoclusters on tribiological properties of dental resin composites. J. Nanotechnol..

[99] Jansto S.G. (2018). The integration of process and product metallurgy in niobium bearing steels. Metals.

[100] Leitune V.C., Takimi A., Collares F.M., Santos P.D., Provenzi C., Bergmann C.P., Samuel S.M. (2013). Niobium pentoxide as a new filler for methacrylate-based root canal sealers. Int. Endod. J..

[101] Leitune V.C., Collares F.M., Takimi A., de Lima G.B., Petzhold C.L., Bergmann C.P., Samuel S.M. (2013). Niobium pentoxide as a novel filler for dental adhesive resin. J. Dent..

[102] Balbinot G.S., Leitune V.C.B., Ogliari F.A., Collares F.M. (2020). Niobium silicate particles promote in vitro mineral deposition on dental adhesive resins. J. Dent..

[103] Garcia I.M., Leitune V.C.B., Balbinot G.S., Balhaddad A.R.A., Melo M.A.S., Samuel S.M.W., Collares F.M. (2021). Physicochemical Effects of Niobic Acid Addition Into Dental Adhesives. Front. Mater..

[104] Crystal Y.O., Niederman R. (2019). Evidence-Based Dentistry Update on Silver Diamine Fluoride. Dent. Clin. N. Am..

[105] Cheng L., Zhang K., Weir M.D., Liu H., Zhou X., Xu H.H. (2013). Effects of antibacterial primers with quaternary ammonium and nano-silver on Streptococcus mutans impregnated in human dentin blocks. Dent. Mater..

[106] Zhang K., Melo M.A., Cheng L., Weir M.D., Bai Y., Xu H.H. (2012). Effect of quaternary ammonium and silver nanoparticle-containing adhesives on dentin bond strength and dental plaque microcosm biofilms. Dent. Mater..

[107] Nyvad B., Crielaard W., Mira A., Takahashi N., Beighton D. (2013). Dental caries from a molecular microbiological perspective. Caries Res..

[108] Garcia I.M., Leitune V.C.B., Takimi A.S., Bergmann C.P., Samuel S.M.W., Melo M.A., Collares F.M. (2020). Cerium Dioxide Particles to Tune Radiopacity of Dental Adhesives: Microstructural and Physico-Chemical Evaluation. J. Funct. Biomater..

[109] Garcia I.M., Leitune V.C.B., Ferreira C.J., Collares F.M. (2018). Tantalum oxide as filler for dental adhesive resin. Dent. Mater. J..

[110] Martins G.C., Meimer M.M., Loguercio A.D., Cecchin F., Gomes O.M.M., Reis A. (2013). Effects of zirconia nanoparticles addition to experimental adhesives on radiopacity and microhardness. Braz. J. Oral Sci..

[111] Zhou H., Weir M.D., Antonucci J.M., Schumacher G.E., Zhou X.D., Xu H.H. (2014). Evaluation of three-dimensional biofilms on antibacterial bonding agents containing novel quaternary ammonium methacrylates. Int. J. Oral Sci..

